# A team science approach for the preclinical and clinical characterization and biomarker development for post‐traumatic epilepsy

**DOI:** 10.1002/epi4.12745

**Published:** 2023-05-10

**Authors:** Sloka S. Iyengar, Laura S. Lubbers, Lauren Harte‐Hargrove, CURE Epilepsy Post‐Traumatic Initiative Advisors, and Investigators, Severn B. Churn, Douglas Coulter, Amanda Hunt, Jaideep Kapur, Daniel Lowenstein, Renee Hebert Martin, James McNamara, Nsini Umoh, H. Steve White, Vicky Whittemore, David W. Wright, Brian Bacskai, Michael Bambrick, Jack L. Browning, Mitchell Butler, Rona Carroll, Beth A. Costine‐Bartell, Jared Davis, Rossella Di Sapia, Ann‐Christine Duhaime, Joseph R. Geraghty, Bryan Golemb, Basso E. K. Gudenschwager, James J. Gugger, Mark Johnson, Victoria Johnson, Pavel Klein, Firas Kobeissy, Kyle Lillis, Jeffrey A. Loeb, Biswajit Maharathi, Dzenis Mahmutovic, Luis Martinez‐Ramirez, Yara Mikhaeil‐Demo, Federico Moro, Kieran Normoyle, Michelle L. Olsen, Dipan C. Patel, Rehan Raiyyani, Teresa Ravizza, Massimo Rizzi, Stefanie Robel, Anna Serafini, Oleksii Shandra, Harald Sontheimer, Kevin Staley, William Stewart, Delia M. Talos, Fernando Testai, Michelle Theus, Alexandra Ulyanova, Pamela VandeVord, Annamaria Vezzani, Troy Volanth, Amy K. Wagner, Kevin K. W. Wang, John Wolf, Jensen Wong, Elisa Zanier

**Affiliations:** ^1^ CURE Epilepsy Chicago Illinois USA

**Keywords:** CURE Epilepsy, post‐traumatic epilepsy (PTE), seizures, team Science, traumatic brain injury (TBI)

## Abstract

**Objective:**

Post‐traumatic epilepsy (PTE) is an acquired epilepsy that develops in the months or years following a traumatic brain injury (TBI) and can lead to substantial personal, financial, and societal burden. To date, PTE is rarely curable; current treatments are partially effective and often accompanied by adverse side effects. While research on PTE has expanded significantly in the last several years, there remain numerous challenges to identifying effective prevention and treatment strategies. In this paper, we describe advances from the CURE Epilepsy PTE Initiative, including its implementation and the emphasis on team science.

**Methods:**

The CURE Epilepsy PTE Initiative funded six research teams to link preclinical and clinical studies to engage in the validation of experimental models, characterization of pathophysiology and biological pathways, and identification of risk factors associated with PTE. Three teams had projects with both a preclinical and a clinical component; these teams focused on: targeting the epileptogenic effects of subarachnoid blood, exploring the neuropathological mechanisms of epileptogenesis, and defining the role of extracellular matrix injury. Two teams undertook entirely preclinical projects: exploring the role of vascular injury, gliosis, and neurogenesis as drivers for PTE, and identifying genetic, proteomic, metabolomic, and microRNA biosignatures to improve the prediction of PTE. One team's project was entirely clinical and investigated genetic and protein biomarkers to improve the prediction of PTE.

**Results:**

In addition to scientific discoveries including characterization of a variety of animal models and progress towards the understanding of biological underpinnings and biomarkers for PTE, significant programmatic and personnel‐related processes were incorporated, including standardized, rigorous policies and procedures to ensure quality and accountability between and within groups.

**Significance:**

We propose CURE Epilepsy's team science approach as an effective way to bring together a diverse set of investigators to explore biological mechanisms that may lead to cures for the epilepsies.

**Abbreviations**
CCI: Controlled cortical impact
COVID‐19: Coronavirus disease
CSF: Cerebrospinal fluid
DoD: Department of Defense
EAC: External advisory council
EEG: Electroencephalography
EpiBioS4Rx: Epilepsy Bioinformatics Study for Antiepileptogenic Therapy
IS: Infantile spasms
KOL: Key opinon leader
MAESC: Mid‐Atlantic Epilepsy and Sleep Center
MRI: Magnetic resonance imaging
P30: Postnatal day 30
PI: Principal investigator
PTE: Post‐traumatic epilepsy
RFA: Request for applications
SAH: Subarachnoid hemorrhage
sGAGs: Sulfated glycosaminoglycans
TAPTE: Team Approach to the Prevention and Treatment of Post‐Traumatic Epilepsy
TBI: Traumatic brain injury

**Key Points**

- Post‐traumatic epilepsy (PTE) develops weeks, months, or years after a traumatic brain injury (TBI) and is associated with a substantial burden to patients and their families and caregivers.
- CURE Epilepsy leveraged a team science initiative infrastructure to investigate the biological mechanisms underlying PTE, laying the groundwork for the development of eventual preventive therapies.
- The goal of the Initiative was to establish a multicenter, multi‐investigator research team focused on PTE that would translate patient‐relevant findings at the molecular, cellular, and systems levels into prevention strategies and/or novel therapies for PTE.
- Six teams working on diverse aspects of PTE pathophysiology were funded over 5 years, and processes for rigorous and standardized protocols and data sharing were instituted.
- As a result of CURE Epilepsy's PTE Initiative, 18 scientific abstracts, 10 conference presentations, seven peer‐reviewed articles, four investigator's workshops, and one data blitz presentation were highlighted at national and international conferences.

## 1 INTRODUCTION

Being diagnosed with post‐traumatic epilepsy on top of recovering from his combat injury was the ultimate challenge. As Pat's brain struggled to heal, frequent seizures rushed in slowing and possibly impacting his overall recovery… Post‐traumatic epilepsy research is the only way to prevent the onset of seizures and spare our service members with traumatic brain injury and their families the additional burden of this life‐threatening disease. –Patty Horan; caregiver to Captain Patrick Horan, Former U.S. Army Captain

Post‐traumatic epilepsy (PTE) is acquired from biomechanical injury to the brain as a result of traumatic brain injury (TBI). Seizures that are an acute manifestation of a head injury occurring within the first week after injury are by convention considered provoked, potentially reflecting reversible perturbation from the recent injury. Recurrent, unprovoked seizures that occur more than a week after the initial injury are considered PTE.^1^ TBI accounts for 20% of clinically symptomatic epilepsy cases and 5% of all epilepsy cases.^2^ The pathophysiology of PTE is complex with many mechanisms at play including neuronal degeneration, vascular dysfunction, and inflammation.^3^ Currently, there are a few known risk factors for PTE such as the severity of the initiating event, but techniques for better prediction, especially in high‐risk individuals are urgently needed.^4^ The latent period between the insult and epilepsy onset presents a unique window of opportunity for epileptogenesis treatment in high‐risk patients; however, at present, no preventive or disease‐modifying therapies are available.

There are many challenges to advancing basic PTE research to improve clinical care. Clinically relevant PTE models have been developed yet no one preclinical model fully recapitulates every characteristic of clinical epilepsy. Translating findings between preclinical and clinical disciplines has been historically difficult owing to the heterogeneous nature of TBI.^5^ While there is some agreement that more severe TBI and specific injury types carry with them a higher likelihood of epilepsy, the effects of milder forms of TBI are unclear.^5^, ^6^ As yet, the study of other risk factors and biomarkers of PTE is still in its early stages, and there are no objective measures to predict which individuals with TBI will develop PTE.^7^ For example, while post‐traumatic epileptiform activity in the brain may precede epilepsy by weeks to months,^7^, ^8^ actual electroencephalogram (EEG) markers of epileptogenesis have been difficult to characterize within the usual brief period of EEG monitoring. Overall, research regarding PTE biomarkers is gaining traction, and some granularity for potential biomarkers has been identified, but more systematic and larger studies are needed to parse out reliable biomarkers and/or pathophysiologic insights for PTE that could lead to potential improvements in prevention or treatment.^9^, ^10^, ^11^

Given the challenges that PTE research has faced, collaborative efforts are imperative to accelerate progress in this area of study. An emphasis on standardization of efforts across scientists and clinicians, and validation of results in the clinic can promote the development of specific treatments for PTE. Thus, initiatives that bring together a diverse group of scientists to address epileptogenesis are critical. To this end, the Epilepsy Center Without Walls Epilepsy Bioinformatics Study for Antiepileptogenic Therapy (EpiBioS4Rx) aims to discover and validate biomarkers of epileptogenesis using both preclinical studies in an animal model of PTE and clinical studies of individuals following TBI.^12^, ^13^ In the European Union, the EPITARGET consortium was created to develop combinatorial approaches to develop effective antiepileptogenic therapies more broadly.^14^ CURE Epilepsy, with its goal of funding basic and clinical research to understand and find disease‐modifying therapies for the epilepsies, has a history of promoting team science to accelerate research discovery.^15^ A previous initiative of CURE Epilepsy – the Infantile Spasms (IS) Initiative – brought together eight teams and provided a unique opportunity for transparent and real‐time collaboration.^16^ To build on the success of the IS Initiative team science approach, the PTE Initiative, funded by the Department of Defense (DoD) convened a group of scientists to leverage teams' expertise and advance the understanding of the pathophysiology and risk factors of PTE. The goals of the Initiative were to develop and validate animal models of PTE, characterize human populations who have sustained TBI and are at risk for PTE, understand PTE risk factors and pathophysiology, and identify PTE biomarkers.

## 2 MATERIALS AND METHODS

### 2.1 Convening of key opinion leaders (KOLs) and development of requests for applications (RFA)

A group of KOLs was convened to define rich areas of scientific opportunity for which a focused, team‐oriented endeavor could significantly impact preclinical and clinical outcomes for people with epilepsy. This group was comprised of subject matter experts in preclinical and clinical TBI, CURE Epilepsy representatives, individuals impacted by PTE, statisticians, and DoD representatives. This group of KOLs was tasked with developing the Initiative's RFA. The primary areas of research addressed in the RFA were (1) to characterize and validate animal models used to study PTE and support the development of novel animal models, and (2) to conduct clinical phenotyping of patients who developed PTE. A subset of the KOLs remained as part of the External Advisory Council (EAC).

### 2.2 Choosing projects and teams

**FIGURE 1:**
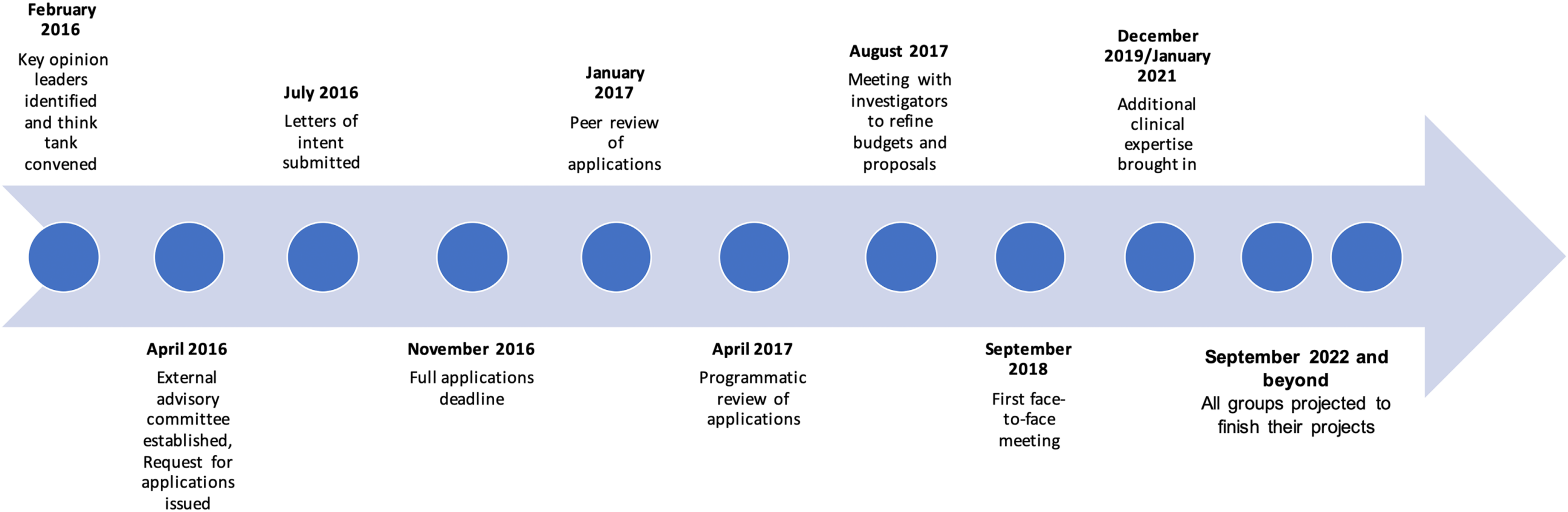
Timelines of the various steps involved in the PTE Initiative, starting from the identification of key opinion leaders and convening of a think tank in February 2016.

**FIGURE 2:**
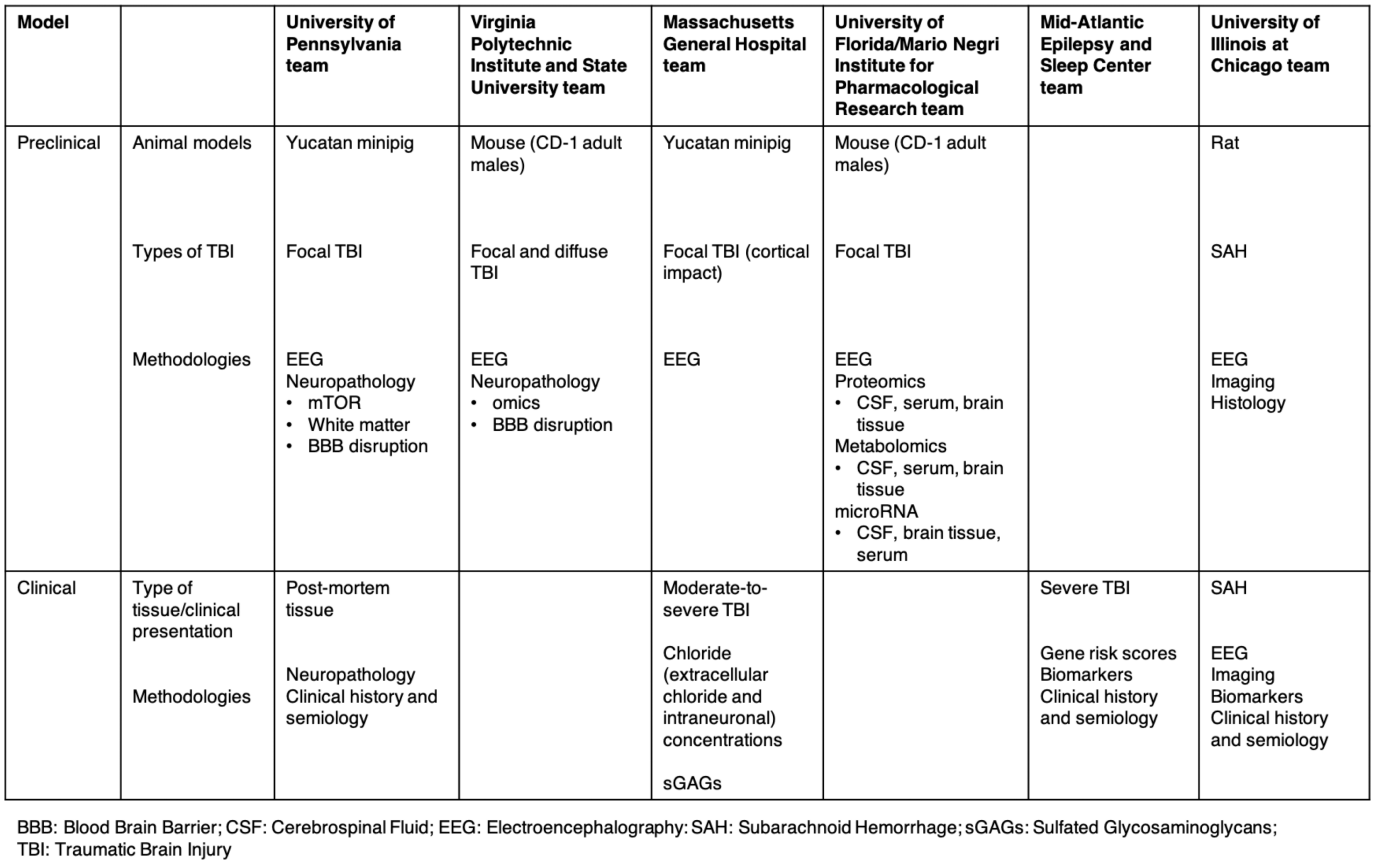
Models of TBI employed and methodologies used by teams to study PTE.

A total of 54 letters of intent were received and reviewed by EAC members and 14 full proposals were ultimately selected for further consideration. After a programmatic review of the research groups and synergies, four projects were initially chosen for inclusion in the PTE Initiative. Preclinical studies were first selected, and clinical projects were wait‐listed for future consideration. Once preclinical projects were initiated, the overall composition of the teams and their objectives were reassessed by CURE Epilepsy staff and the EAC. Gaps in addressing the intent of the RFA were identified and two of the wait‐listed clinical studies that filled those gaps and aligned with the preclinical projects were funded. A total of six teams were funded over 5 years, and the combination and synergy of preclinical and clinical approaches emerged as a main success of the PTE Initiative. PTE Initiative teams and collaborators were located within the United States and internationally, enabling a global collaboration. Figure 1 provides an overview of the timelines and steps of the Initiative, and Figure 2 provides an overview of the projects, experimental models used, and methodologies employed for each team.

### 2.3 Predetermined metrics of success

PTE Initiative teams, CURE Epilepsy staff, and the EAC developed measures of success, which were used to track the scientific goals of the teams. These were to:

- Develop animal models that align with human PTE.
- Identify PTE biomarkers to understand the pathophysiology and pathways after TBI that may lead to PTE. For this Initiative, a biomarker was defined as a variable that can be measured in a variety of ways, for example, through genomic analysis, in blood, or via brain activity to indicate the risk of PTE.
- Provide insight into the role of astrocytes vs. neurons in the progression of PTE.
- Provide a deeper understanding of focal vs. diffuse mechanisms of injury following TBI.
- Provide a better understanding of vascular integrity, neurogenesis, and EEG signatures of PTE.

These metrics were used as guiding principles rather than strict guidelines to keep sight of the ideal end goals of the Initiative while acknowledging that a key principle of the Initiative was letting the science guide the projects. Hence, there were yearly conversations on whether or not the Initiative's self‐defined overarching goals were on track; if goals were not met, CURE Epilepsy staff, Advisors, and members of the teams troubleshot ways to achieve specified goals. Administratively, a success criterion was to create a structure for data standardization and transparency via the creation of data standardization tools.

## 3 RESULTS

### 3.1 Structure of the PTE initiative

The overarching philosophy of the PTE Initiative was to allow scientific nimbleness for teams so they could explore promising results and adapt their strategy in real‐time. Scientific goals and target timelines were defined during the contracting process, before work began, and were integral to tracking the progress of each project. However, there was a certain degree of scientific flexibility. Hence, while teams were encouraged to investigate promising findings, there was the ability to end portions of projects that were not going as planned or producing results so as not to waste resources.

While quarterly reports and meetings were mandated by the DoD, other aspects of the Initiative were co‐created with CURE Epilepsy in response to the teams' needs and perspectives as the Initiative progressed. The latter included auditing processes, the involvement of two different advisory panels, policies, an extensive meeting structure to encourage collaboration, early career investigator involvement, and data‐sharing efforts. Specific details about personnel, policies, reporting and auditing, data curation and storage, and progress meetings are provided in Supporting Information S1.

### 3.2 Scientific outcomes

The teams worked on preclinical and clinical themes and scientific topics (Figure 2, Table 1). Several scientific successes arose from the PTE Initiative; these include the development and validation of mouse models and novel porcine models for studying PTE and a focus group to investigate changes in EEG signatures in human and experimental PTE. At the time of this manuscript, 18 scientific abstracts, 10 conference presentations, seven peer‐reviewed articles, four investigator's workshops, and one data blitz presentation were presented nationally and internationally (Table 2).

**TABLE 1:** Specific aims of projects funded as part of the PTE initiative.

Institute	PI	Title of the project	Aims of the project
Projects with preclinical and clinical aspects			
University of Pennsylvania	Victoria E. Johnson, MBChB, Ph.D.	Neuropathological mechanisms of epileptogenesis in PTE	Aim 1: Determine the relative frequency of focal vs. diffuse injuries following TBI by examining humans with and without PTE vs. controls Aim 2: Determine the role of persistent BBB dysfunction in epileptogenesis after TBI in humans and porcine models Aim 3: Determine the role of glial pathology and mTOR activation in epileptogenesis after TBI in humans and porcine models
Massachusetts General Hospital	Kevin Staley, MD	The role of extracellular matrix injury in PTE	Aim 1: Investigate whether intracellular and extracellular chloride concentrations are altered in regions of experimental brain injury and MRI diffusion changes validating the P30 piglet CCI model of PTE and developing an optical system for measurement of intra/extracellular chloride concentrations Aim 2: Investigate whether CSF sGAGs levels are a candidate biomarker for human brain and matrix injury by measuring CSF sGAGs levels in brain‐injured human patients and by measuring CSF sGAGs levels in brain‐injured piglets
The University of Illinois at Chicago team	Jeffrey Loeb, MD, Ph.D.	Targeting epileptogenic effects of subarachnoid blood in TBI	Aim 1: Identify patients with subarachnoid hemorrhage (SAH) and utilize early EEG, imaging, laboratory, and clinical findings to identify factors that predict who develops epilepsy following SAH Aim 2: Identify biomarkers of epileptogenesis utilizing EEG, imaging, and behavioral quantitation in a rat model of SAH Aim 3: Investigate common EEG biomarkers for SAH in human and animal models utilizing shared EEG data
Projects with only preclinical aspects			
Virginia Polytechnic Institute and State University	Harald Sontheimer, Ph.D. and Michelle Olsen, Ph.D.	Vascular injury, gliosis & neurogenesis as drivers for PTE	Aim 1: Comparatively assess the CCI and repeated diffuse TBI (closed‐head impact‐acceleration) models to characterize seizure onset, incidence, frequency, and EEG properties when injury type, frequency, and severity are varied. Determine if a predictive EEG characteristic occurs during the latent period that is indicative of the onset of spontaneous seizures in each model Aim 2: Define the cellular, molecular and functional changes that differentiate the animals that develop PTE from those that do not
University of Florida/Mario Negri Institute for Pharmacological Research	Kevin Wang, Ph.D.	Identifying proteomic, metabolomic, and microRNA biosignatures for spontaneous PTE in a CD1 mouse model	Aim 1: Conduct CCI in a cohort of mice and use video monitoring and EEG assessment to identify subgroups of mice with [Seizure (+)] and without [Seizure (−)] spontaneous seizures at 5 months postinjury and collect biosamples (brain tissue, CSF, serum) Aim 2: Perform global and targeted proteomic analysis on the cortex, hippocampus, and striatum lysate and biofluid samples from a subset of post‐CCI Seizure (+) and Seizure (−) mice at 5 months postinjury Aims 3,4: Perform targeted metabolomic analysis and targeted microRNA on the cortex, hippocampus, and striatum lysate and biofluid samples from a subset of post‐CCI Seizure (+) and Seizure (−) mice at 5 months postinjury Aim 5: Analyze additional samples from other PTE Initiative groups for proteomic, metabolomic, and miRNA profile verification
Project with only clinical aspects			
Mid‐Atlantic Epilepsy and Sleep Center	Pavel Klein, MD	Genetic and protein biomarkers of PTE to improve prediction of PTE: a prospective study in an enriched patient population	Identify genetic and plasma biochemical biomarkers of prediction of PTE Genetic factors include specific single nucleotide polymorphisms in genes associated with adenosine metabolism, glutamate transporters, and IL‐1β in TBI subjects who do vs. who do not develop PTE Biochemical factors include high mobility group box‐1 protein (HMGB1) and cytokine interleukin (IL)‐1β

Abbreviations: BBB, Blood–Brain Barrier; CCI, Controlled Cortical Impact; CSF, Cerebrospinal Fluid; EEG, Electroencephalography; MRI, Magnetic Resonance Imaging; mTOR, Mammalian Target of Rapamycin; P30, Postnatal Day 30; SAH, Subarachnoid Hemorrhage; sGAGs, Sulfated Glycosaminoglycans; TBI, Traumatic Brain Injury.

**TABLE 2:** List of publications, abstracts, conference presentations, grants, and submitted grant applications as a result of the CURE Epilepsy PTE Initiative.

University of Pennsylvania team Conference abstracts Ulyanova A, Hamid H, Omole A, Chen HI, Johnson VE, Wolf JA. Sleep disturbances as a potential mechanism of post‐traumatic epileptogenesis. Annual Meeting of the American Epilepsy Society, 2021. Chicago, Illinois. Ulyanova A, Cottone C, Litt B, Chen HI, Johnson VE, Wolf JA. A translational model of post‐traumatic epileptogenesis. 37th Annual National Neurotrauma Symposium, 2019. Pittsburgh, Pennsylvania. Data blitz Hanlon LA, Stewart W, Atkinson J, Wolf JA, Jensen F, Talos D, Johnson VE. mTOR pathway activation implicated in epileptogenesis after TBI. 37th Annual National Neurotrauma Symposium, 2019. Pittsburgh, Pennsylvania. Conference presentations Johnson V. Neuropathological Mechanisms of Epileptogenesis in Post‐traumatic epilepsy. 38th Annual National Neurotrauma Symposium, 2021. Virtual conference. Wolf, JA. Modeling Post‐Traumatic Epilepsy in the Gyrencephalic Brain, 39th Annual National Neurotrauma Symposium, 2022. Atlanta, Georgia.
Massachusetts General Hospital team Conference abstracts Normoyle KP, Dzhala V, Lillis K, Egawa K, Glykys J, Rahmati N, Staley K. Brain extracellular matrix alters local ion concentrations and responses to injury. Annual Meeting of the American Epilepsy Society, 2021. Chicago, Illinois. Ulyanova A, Cottone C, Litt B, Chen HI, Johnson VE, Wolf JA. A translational model of post‐traumatic epileptogenesis. 37th Annual National Neurotrauma Symposium, 2019. Pittsburgh, Pennsylvania. Lillis K, Costine‐Bartell B, Martinez‐Ramirez L, Normoyle, KP, Staley KJ. Two‐photon imaging of intra‐ and extracellular chloride in a porcine model of post‐traumatic epilepsy. Society for Neuroscience Annual Meeting, 2021. Virtual conference. Raiyyani R, Martinez‐Ramirez L, Duhaime AC, Staley KJ, Costine‐Bartell B. Porcine post‐traumatic porcine epilepsy: Semiology. Annual Meeting of the American Epilepsy Society, 2020. Virtual conference. Lillis K, Lau LA, Balena T, Staley KJ. Absence of neuronal death during status epilepticus in vitro. Annual Meeting of the American Epilepsy Society, 2020. Virtual conference. Martinez‐Ramirez L, Raiyyani R, Price G, Zhao J, Ding A, Duhaime AC, Staley, KJ, and Costine‐Bartell B. Development of seizures in a large animal model of post‐traumatic epilepsy. Annual Meeting of the American Epilepsy Society, 2020. Virtual conference. Lillis K, Lau LA, Staley KJ. Imaging the Emergence of Interictal Spikes in Post‐Traumatic Epilepsy. Annual Meeting of the American Epilepsy Society, 2019. Baltimore, Maryland.
Normoyle KP, Dzhala V, Lillis K, Egawa K, Glykys J, Rahmati N, Staley KJ. Brain Extracellular Matrix Alters Local Ion Concentrations and Responses to Injury. Society for Neuroscience Annual Meeting, 2019. Chicago, Illinois. Conference presentation Lillis K. Two‐photon imaging of intra and extracellular chloride in a porcine model of post‐traumatic epilepsy. Annual Meeting of the American Epilepsy Society, 2021. Chicago, Illinois. Publications Release of extracellular matrix components after human traumatic brain injury. To be submitted to J Neurotrauma. Martinez‐Ramirez L, Slate A, Price G, Duhaime AC, Staley K, Costine‐Bartell BA. Robust, long‐term video EEG monitoring in a porcine model of post‐traumatic epilepsy. eNeuro. 2022;9(4): ENEURO.0025–22.2022. doi:10.1523/ENEURO.0025‐22.2022. Investigators' Workshop Inhibitory plasticity, ictogenesis, and epileptogenesis. To be presented at the Annual Meeting of the American Epilepsy Society, 2022. Nashville, Tennessee.
The University of Illinois at Chicago team Conference abstracts Maharathi B, Wong, J, Geraghty, JR; Serafini, A; Davis, J, Butler, M, Kolar SK, Pandey DK, Loeb J. Multimodal data integration platform combining clinical and preclinical models of post subarachnoid hemorrhage epilepsy. 44th Annual International Conference of the IEEE Engineering in Medicine & Biology Society (EMBC); 2022; Glasgow, UK Geraghty JR, Butler MP, Loeb JA, Testai FD. Immunomodulation with fingolimod reduces microglial responses and improves neurologic outcome after experimental subarachnoid hemorrhage. Chicago Society for Neuroscience, 2021. Chicago, Illinois. Butler MP, Geraghty JR, Sudhakar D, Lung TJ, Testai FD, Loeb JA. An MRI‐based method for assessing the extent and location of bleeding in a rat model of subarachnoid hemorrhage. International Stroke Conference 2021. Virtual conference. Geraghty JR, Maharathi B, Butler MP, Lung TJ, Balas G. An animal model of post subarachnoid hemorrhage epileptogenesis shows parallels to human EEG and implicates neuroinflammation as a pathogenic mechanism. Annual Meeting of the American Epilepsy Society, 2020. Virtual conference. Geraghty JR, Katz EA, Maharathi B, Dachet F, Testai FD, Loeb JA. Neuroinflammation after subarachnoid hemorrhage as a driver of epileptic networks. Gordon Research Conference: Neuroimmune Communication in Health & Disease, 2019. Ventura, California. Geraghty JR, Maharathi B, Muhammad A, Xu H, Loeb JA, Testai FD. Early brain injury after subarachnoid hemorrhage and its potential role in epileptogenesis: animal models and therapeutic applications. International Stroke Conference, 2018. Los Angeles, California.
Virginia Polytechnic Institute and State University team Conference abstracts Shandra O, Mahmutovic D, Maharathi B, Loeb J, Robel S. Traumatic brain injury causes atypical astrocytes and dynamic spectral alterations on EEG. Annual Meeting of the American Epilepsy Society, 2021. Chicago, Illinois. Investigators' Workshop Shandra O. Cellular and electrographic signatures of post‐traumatic epileptogenesis after brain injury. Annual Meeting of the American Epilepsy Society, 2021. Chicago, Illinois. Browning J, Gudenschwager Basso EK, Sontheimer H, Robel S, VandeVord P, et al. Unique hilus astrocyte morphology and gliosis following CCI brain injury: A predictive phenotype for post‐traumatic epilepsy? Central Virginia Society for Neuroscience Meeting, 2022. Charlottesville, Virginia. Publications Shandra O, Maharathi B, Mahmutovic D, Robel S. Early and late onset changes in the EEG power spectrum after diffuse traumatic brain injury as markers of post‐traumatic epileptogenesis. In preparation. Shandra O, Robel S. Inducing post‐traumatic epilepsy in a mouse model of repetitive diffuse traumatic brain injury. J Vis Exp. 2020;(156):10.3791/60360.
University of Florida/Mario Negri Institute for Pharmacological Research team Conference abstract Di Sapia R, Moro F, Tolomeo D, Micotti E, Ravizza T, Vezzamo AM. A newly refined mouse model of post‐traumatic epilepsy for biomarker and drug discovery. Annual Meeting of the American Epilepsy Society, 2019. Baltimore, Maryland. Conference presentations Speaker: Zanier E. Modeling post‐traumatic epilepsy for therapy development: How it started, how it's going. In IRFMN, CURE Epilepsy Frontiers in Research Seminar Series, 2022. Departments of Emergency Medicine, Neurology and Neuroscience, University of Florida College of Medicine. Chair: Harte‐Hargrove, L; co‐chair: Kobeissy F. Speakers: Zanier E, Duncan D, Pitkanën A, Wolf J. Current status of animal models of post‐traumatic epilepsy (PTE): The need for true translational models of PTE. 39th Annual National Neurotrauma Symposium, 2022. Atlanta, Georgia. Speaker: Zanier E. CD1 Mouse as a novel model for post‐traumatic epilepsy: omics and biomarker discovery. 39th Annual National Neurotrauma Symposium, 2022. Atlanta, Georgia. Speaker: Wang K. Acute and post‐acute prognostic biomarkers after TBI. 39th Annual National Neurotrauma Symposium, 2022. Atlanta, Georgia. Co‐chairs: Wang K and Harte‐Hargrove L. Post‐Traumatic Epilepsy: TBI and epilepsy crosstalk studies. 38th Annual National Neurotrauma Symposium, 2021. Virtual conference. Speakers: Bartell B, Lillis K, Ramirez LM, Raiyyani R, Normoyle K et al. Do changes in the brain's extracellular matrix contribute to post‐traumatic epilepsy? Theus M. Vascular injury, gliosis and neurogenesis as drivers of post‐traumatic epilepsy. Wagner A, Awan N, Fan E, Linsenmeyer M, Conley Y, Grafman J. Genomic risk factors in post‐traumatic epilepsy. Speaker: Kobeissy F. Identifying proteomic, metabolomic and microRNA signatures for spontaneous seizures in a CD1‐mouse model TBI. 38th Annual National Neurotrauma Symposium, 2021. Virtual conference. Co‐chairs: Menon D and Wang K, Emerging trends in molecular TBI biomarkers. 38th Annual National Neurotrauma Symposium, 2021. Virtual conference. Co‐chairs: Schober S and Robertson C. Neurological manifestations of COVID‐19 and potential overlaps with neurotrauma. 38th Annual National Neurotrauma Symposium, 2021. Virtual conference. Publications Jalloul D, Hajjar H, Asdikian R, Maawie M, Nasrallah L, Medlej R, Darwich M, Karnib N, Lawand N, Rassoul RA, Wang KKW, Kobeissy F, Darwish H, Obeid. Potentiating hemorrhage in a periadolescent rat model of closed‐head traumatic brain injury worsens hyperexcitability but not behavioral deficits. Int J Mol Sci. 2021;22(12):6456. Di Sapia R, Moro F, Montanarella M, Iori V, Micotti E, Tolomeo D, Wang KKW, Vezzani A, Ravizza T, Zanier ER. In‐depth characterization of a mouse model of post‐traumatic epilepsy for biomarker and drug discovery. Acta Neuropathol Commun. 2021; 26;9(1):76. Simonato M, Agoston DV, Brooks‐Kayal, A. Dulla C, Fureman B, Henshall DC, Pitkänen A, Theodore WH, Twyman RE, Kobeissy FH, Wang KK, Whittemore V, Wilcox KS. Identification of clinically relevant biomarkers of epileptogenesis — a strategic roadmap. Nat Rev Neurol, 2021;17: 231–242.
CURE Epilepsy Presentations Investigator's Workshop Moderator: Harte‐Hargrove L. Speakers: Moro F, Shandra O, Gugger JJ, Lillis K. Novel Techniques and Models to Identify Epileptogenesis in Post‐traumatic Epilepsy. Annual Meeting of the American Epilepsy Society, 2021. Chicago, Illinois.

One of the major benefits of CURE Epilepsy's team science approach was the collaborations that came about as a result of the Initiative; some collaborations were spontaneous, and others were explicitly encouraged by CURE Epilepsy. Group meetings where all teams were present were the primary opportunity for crosstalk and exploratory conversations on findings in the animal models as they applied to the heterogenous human condition of PTE. Serendipitous collaborations between teams and outside groups were also formed; for example, an outside group with expertise in machine learning and artificial intelligence joined the EEG focus group and presented ideas to refine EEG analysis. Research projects were intentionally chosen because of similar or complementary models as well as the ability to share unique technology that might complement another team; for this reason, synergy was achieved on a variety of levels, as described below.

#### 3.2.1 University of Pennsylvania (topic: Exploring the neuropathological mechanisms of epileptogenesis)

The University of Pennsylvania team used post‐mortem human tissue and porcine controlled cortical impact (CCI) model of TBI that recapitulates both focal and diffuse features of human TBI to explore potential neuropathological mechanisms associated with the development of PTE following TBI. To describe the nature and distribution of pathology, the team performed a wide range of immunohistochemistry on tissue from Yucatan minipigs examining blood–brain barrier (BBB) dysfunction, glial pathology, and mammalian target of rapamycin (mTOR) activation following TBI. To characterize the model and utilize the broad expertise of the PTE Initiative team members, porcine EEG data were discussed during the Initiative's EEG focus group meetings. To complement this work and explore the clinical relevance of their model, the team performed parallel examinations on post‐mortem samples from humans with a history of TBI and PTE, sourced from the Glasgow TBI Archive within the Department of Neuropathology, Queen Elizabeth University Hospital, Glasgow, UK. The team's preliminary data suggest activation of the mTOR pathway following TBI in humans.

#### 3.2.2 Massachusetts General Hospital (topic: Defining the role of extracellular matrix injury)

The Massachusetts General Hospital team developed a dual‐contusion model of PTE in Yucatan minipigs, with initial data suggesting an increase in epileptiform discharges around the time of onset of convulsions.^17^, ^18^ Preliminary data from this model also suggests approximately 50% incidence of PTE over several months and explored a working sequence of peri‐ictal behaviors of PTE in this large animal model.^19^ Specifically, the team described the pattern of simple and complex spikes, spike trains and convulsions, accompanied by disturbed sleep patterns in the minipigs.^18^ In addition, the team developed in vivo cortical chloride imaging to test the hypothesis that damage to the extracellular matrix may play a role in the development of PTE following TBI and that this can be evidenced by changes in extracellular and intracellular concentrations of chloride levels. The team's preliminary investigation suggests an acute increase in extracellular and intraneuronal concentrations of chloride hours after cortical impact followed by a chronic decrease in extracellular chloride at the site of impact, measured >1 year postinjury.^17^

In addition to their porcine model, the team also examined individuals with moderate–severe TBI, investigating cerebrospinal fluid (CSF), blood, and urine sulfated glycosaminoglycans (sGAGs) levels as a candidate biomarker for human brain and extracellular matrix injury. The COVID‐19 pandemic brought with it unique challenges to the team's goal of recruiting 28 individuals for the study of released sGAGs in comatose head trauma patients. A COVID‐related decrease in enrollment was primarily due to a decrease in TBI (likely due to fewer individuals being active outside of their homes) and a temporary moratorium on nonessential research‐related recruitment to clinical studies. Ultimately, the team was able to recruit and obtain CSF, blood, and urine samples from 14 patients, which was the first study to measure sGAGs in TBI patients and the first to measure them in ventricular fluid. Analysis showed that sGAG levels were not elevated in ventricular fluid, suggesting that they are rapidly metabolized locally in tissues.

#### 3.2.3 University of Illinois at Chicago (topic: Targeting the epileptogenic effects of subarachnoid blood)

The University of Illinois at Chicago team examined patients with subarachnoid hemorrhage (SAH) and a rat model of SAH caused by endovascular perforation, to utilize magnetic resonance imaging (MRI) and EEG to characterize biomarkers of the development of PTE. The team recruited a total of 162 individuals with SAH who presented at the University of Illinois at Chicago Hospital between 2010 and 2020, collecting all available data from the intensive care unit including serial brain computed tomography scans and EEG data for biomarker assessment. Recruitment involved contacting over 700 potential patients by phone and letter, a process made more difficult by COVID‐19 institutional research closures. The team also developed a web‐based application that supports data visualization, downloading, and sharing to model multimodal longitudinal data from both humans and rodents to analyze and compare potential risk factors for PTE over time.^20^ Pointing to cross‐team synergies, the team's algorithms for EEG analysis were used to support the cross‐comparison of CCI data from the University of Florida and Mario Negri Institute for Pharmacological Research teams. The University of Illinois at Chicago team shared their algorithms for seizure detection and spectral analysis with the Virginia Polytechnic Institute and State University team, where the algorithms were used to validate seizures in their models and further optimize it in collaboration with The University of Illinois at Chicago team.

#### 3.2.4 Virginia Polytechnic Institute and State University (topic: Understanding the role of vascular injury, gliosis, and neurogenesis as drivers for PTE)

Utilizing a mouse CCI model with a focal injury in addition to a mouse weight drop model with a more diffuse injury, the team focused on classifying changes in EEG, the number, structure, and location of neuronal support cells and alterations in the astrocyte transcriptome that might lead to the development of PTE following TBI. The team was initially challenged in achieving the required rate of PTE in both models due to several variables, including seizures in sham animals from a particular vendor, a greater‐than‐expected exclusion of animals, and experimental issues with EEG electrode implantation. Both models were refined to achieve a > 25% PTE+ rate through methodological troubleshooting that included the use of a wild‐type mouse strain that did not exhibit seizures following sham procedures, changes in EEG implantation protocols, and, in the case of the CCI model, the use of different injury severities that greatly benefited from synergistic collaboration with the University of Florida and Mario Negri Institute for Pharmacological Research teams that utilized a similar model.

In developing their weight drop model, the team found that their experimental procedures were sufficient to produce convulsive and nonconvulsive seizures.^21^ Also, similar to the human pathophysiology, a latent period to seizure onset following the injury was found with this modified weight drop model.^21^ EEG from both models was extensively analyzed and discussed with other Initiative teams during EEG focus group meetings, with preliminary results showing significant differences in high‐frequency oscillations and slow frequency power analysis in CCI animals that developed PTE.

#### 3.2.5 The University of Florida (topic: Identifying genetic, proteomic, metabolomic and microRNA biosignatures to improve prediction of PTE)

The University of Florida team used the CCI mouse model of TBI^22^, ^23^ implemented by a teammate at the Mario Negri Institute for Pharmacological Research in Milan, Italy, to examine proteomic, metabolomic and miRNA biosignatures relating to the development of PTE. Proteomics measures the set of proteins in a given cell or tissue at a given moment, metabolomics measures the cell's metabolites, and microRNAs are small noncoding RNA molecules that modulate basic biological processes. Changes in microRNAs have been implicated in certain aspects of epilepsy.^24^ The team found that induction of severe TBI was associated with the development of PTE in 60% of animals.^25^ Preliminary multiomics (proteomics, metabolomics, and lipidomic) analyses showed molecular alterations related to epileptogenesis. These included marked changes in glial fibrillary acidic protein and prominent metabolomic candidates [N‐acetyl‐aspartyl‐glutamate (NAAG) and its hydrolysis product N‐acetyl‐aspartate (NAA)] among TBI and the PTE subject animals. Neurology‐specific microRNAs (mir30D, mir125, and mir21 among others) were shown to be upregulated in the PTE group. These were mapped to specific brain functions relevant to PTE including neuroinflammation. The team also conducted a characterization of electrocorticography during the first 7 days post‐TBI to identify measures predictive of PTE development by the quantification of the epileptiform activity, the analysis of nonlinear dynamics, and spectral analysis. Preliminary data indicated signatures possibly predictive of epileptogenesis following TBI, with final results pending.

#### 3.2.6 Mid‐Atlantic Epilepsy and Sleep Center (MAESC; topic: Investigating genetic and protein biomarkers to improve prediction of PTE)

To investigate possible clinical, genetic, and protein biomarkers of PTE, the MAESC team examined a group of individuals with severe TBI with the intent to follow them clinically for two years. In addition, they planned to prospectively evaluate a set of genetic and protein biomarkers supported by previous animal and/or human studies. Working with a network of 10 clinical sites located within the US and Europe, the team recruited 110 individuals with TBI. This required an approach of maintaining frequent communication with clinical site coordinators to overcome challenges unique to the COVID‐19 pandemic such as regulatory delays, supply chain shortages, a decrease in TBI rate, and challenges with patient follow‐up. Though study analyses will not be completed until the end of the two‐year follow‐up period (estimated to be late 2023), preliminary analyses have begun to parse the clinical characteristics of individuals with TBI/PTE. These analyses include examination of multiomic signatures in human biosamples by the University of Florida team, adding an additional cross‐team point of collaboration and synergy.

#### 3.2.7 Cross‐validation of targets

Cross‐validation of biological targets involved in PTE, in general, is difficult because of the heterogeneity of the condition in human patients and in animal models used to simulate the condition. Hence, cross‐validation of targets was not an explicit goal in the PTE Initiative. However, there were two instances of collaborative cross‐validation and replication: in one instance, methods and results between two teams using the CCI mouse model were examined, resulting in the modification of procedures by one of the teams to align the models to a greater extent. In the second instance, TBI leading to spontaneous seizures in the porcine brain was validated in two models, both of which were the first demonstration of epilepsy following TBI in swine. Some similarities exist between the CURE Epilepsy PTE Initiative and other related projects (e.g., the EpiBioS4RX Study and EPITARGET). For example, these projects were focused on understanding different biological aspects of PTE in animal models and uncovering biomarkers for PTE in humans with TBI. Notably, the CURE Epilepsy PTE Initiative was different from these related projects because characterization of a variety of PTE animal models was an intentional part of this Initiative with the goal of modeling and understanding part of the spectra of clinical TBI cases, whereas these other projects focused on one animal model. Though the study design of the PTE Initiative helps address the gap between preclinical pathophysiology and clinical diagnosis, the accompanying greater potential to have low‐powered studies will necessitate additional follow‐up studies to confirm results.

#### 3.2.8 Focus groups

Focus groups were created after recognizing the need to delve into certain topics on a deeper level. Focus groups met frequently, and early career researchers were encouraged to lead and present at these meetings. These discussions helped advance understanding of concepts, were an opportunity to hone in on specific aspects that may be relevant to each PI's project and the field of PTE and were an opportunity for early career scientists to develop leadership skills. The EEG focus group, data sharing focus group, and the clinical and preclinical common data element (CDE) focus groups were created as part of the Initiative (Supporting Information S2).

### 3.3 Successes of the initiative

#### 3.3.1 Success: Letting the science drive the direction

The PIs noted the impact of the PTE Initiative on their work and their contribution to the field of epilepsy. In Dr. Staley's words, “The PTE Initiative provided funding for a high‐risk, high‐impact study of a potential mechanism for PTE. Our findings have provided key preliminary data that have substantially de‐risked the project.”

According to Dr. Loeb, the sentiment of “letting the science guide the direction” manifested itself in the data. In his words, “It all comes down to data. Critical evaluation of the raw, unedited data to curate real data vs. experimental artifacts enables scientific discovery without bias.” For Dr. Olsen, the structure of the Initiative to let the science guide the direction was helpful as such: “Working with the other investigators has been incredibly useful in refining our models, in that in quarterly meetings we identified similar problems and through a reiterative process worked through those problems.” For Dr. Olsen's team, “letting the science lead the direction” also led to unanticipated discoveries such as unique characteristics that define or stratify changes in astrocytes across animals with and without PTE, identification of unique EEG signatures and sleep EEG patterns that may be unique to mice with PTE, and identification of potential differences in neurogenesis across mice with and without PTE.

#### 3.3.2 Success: Being a part of a bigger team

The collaborative nature of the Initiative provided teams with the ability for cross‐validation. Dr. Staley mentioned, “Our focus had been on gathering preliminary data rather than validation, but the validation is a very useful adjunct to our other findings.” He also mentioned that “a positive aspect of team science was hearing about the experience of another group which approached the…problem from a different perspective.” The collaborative nature also provided advantages in terms of speed. Dr. Zanier who partnered with Dr. Wang from the University of Florida mentioned, “Thanks to CURE Epilepsy support we were able to speed up our studies, but especially we could join a very active consortium aimed at discussing hypotheses, sharing data and cross‐validating results across preclinical models and patients.” On the flip side of this collaborative power was the difficulty inherent in working with other teams. Dr. Loeb commented, “It is sometimes challenging to disagree with other teams on approaches and interpretation of results, but this is very healthy and what is needed in many scientific inquiries.” While some PIs felt that meetings were time‐consuming, Dr. Churn (an advisor of the Initiative) reported that “The multiple PI meetings were essential in building collaborations, harmonizing data, and developing standards that improved each PI's research programs.”

#### 3.3.3 Success: Impact on early career investigators

Early career scientists were given opportunities for growth due to their involvement in focus groups where they were encouraged to present findings. In total, 21 early career investigators were involved in the Initiative. One early career investigator (Dr. Geraghty) mentioned “It is nice to see many groups openly sharing data, collaborating, and leveraging each other's experiences. Being exposed to multiple clinical, translational, and basic science approaches to [study] PTE will undoubtedly be valuable for my future career as I work towards eventually establishing my independent lab.”

### 3.4 Challenges, areas of growth, and learnings from the Initiative

The PTE Initiative revealed several areas of growth in the areas of data sharing and standardization and challenges brought about by the COVID‐19 pandemic. The frequency and cadence of meetings and the perceived effort required for reporting also emerged as challenges. Although reporting was mandated by the DoD and meetings were organized to foster collaboration between the teams, these efforts proved time‐consuming.

#### 3.4.1 Challenge: Data sharing

CURE Epilepsy developed a data‐sharing policy with the teams so that terms would be agreed upon; however, data sharing was a challenge for various reasons. Teams were not explicitly required to share their data with other teams, there was no incentive for data sharing, and there was an additional effort required on the part of the teams to upload the data to a central location. Technical issues were also encountered, for example, the availability of a data‐sharing platform, and logistical issues such as formatting data in a standardized way. Hence, uploading the teams' complex data to a central data repository ended up taking place to a minimal extent. While this was a challenge in the CURE Epilepsy PTE Initiative, the lack of data sharing remains an issue in scientific research in general and reflects a systemic issue. It will likely take concerted efforts from funding agencies to make transparent data sharing a mandated requirement (for example, the requirement for data sharing at the National Institutes of Health that has gone into effect in early 2023). Important learnings from the PTE Initiative include the need for open data ecosystems that allow research to be findable, accessible, interoperable, and reusable,^26^ allowing the research community to aggregate important data for meta‐analysis, and speeding progress towards prevention for PTE. Data sharing as ideated might have led to a bigger impact of the Initiative; still, conversations around data sharing did help build consensus and guidelines around nuances regarding data sharing with individuals outside the Initiative and appropriate citation of team members' data when shared. At the time of this publication, team members are still discussing the ways in which data sharing between teams may happen beyond the scope of the Initiative.

#### 3.4.2 Challenge: Impact of COVID‐19

The COVID‐19 pandemic affected many aspects of the PTE Initiative. Labs were closed and supply chains for resources including experimental animals and equipment were disrupted. During the period of shutdown, teams were able to assign essential personnel to manage animal care, continue EEG analysis and video monitoring, harvest tissues, and complete histology. Though an inability to work on‐site delayed certain aspects of data analysis, this also presented an opportunity to establish best practices for remote data analysis and focus on processing and analyzing already‐collected data.

Administrative processes were also impacted by the COVID‐19 pandemic and particularly meetings were impacted. CURE Epilepsy adapted meetings to a virtual format to ensure opportunities for continued collaboration and problem‐solving. There were certain disadvantages including the lack of spontaneous discussion and collaboration that occurs in an in‐person setting, which are important components for ensuring understanding of concepts for such a multidisciplinary team.

## 4 CONCLUSIONS

The PTE Initiative was based on CURE Epilepsy's IS Initiative and addressed many of the areas of growth that were revealed therein.^16^ The PTE Initiative convened investigators working on varied facets of PTE to develop and validate preclinical models, better understand its pathophysiology, and lay the groundwork for the development of disease‐modifying therapies. In addition to its contributions to the field of PTE, this Initiative continued to demonstrate that a team science approach can be an effective way to make transformative changes in a field of science.

### 4.1 Looking ahead

#### 4.1.1 Revisiting metrics of success and the future of PTE initiative team scientific projects

Teams developed and characterized several different animal models that align at least in part with human PTE. Teams enhanced our understanding of the role of astrocytes vs. neurons in the progression of PTE, while also providing a deeper understanding of focal vs. diffuse mechanisms of injury following TBI through the use of varying preclinical models. Hence, the Initiative led to progress towards metrics of success and defined areas in need of additional study.

The Initiative unearthed several challenges that need additional attention. Though some progress was made in the understanding of potential biomarkers for PTE, the Initiative reinforced that the study of different types of PTE biomarkers is necessary given the complexity of the condition. Mapping the temporal dynamics of these biomarkers and translating them into the clinical setting will prove invaluable to finding eventual therapies for PTE. Particularly challenging was the goal to understand EEG biomarkers of PTE. Measuring, evaluating, and interpreting EEG is essential to understanding possible EEG biomarkers, and these conversations should continue in the future to understand how brain activity changes after TBI, and identify potential EEG biomarkers of PTE. Additionally, ensuring data are accessible and able to be federated for user‐friendly data sharing is essential to progress.

Importantly, at the time of publication, the work of four teams on the PTE Initiative is still ongoing and many teams are pursuing additional funding to continue their research. Specifically, additional analysis of human samples is underway to expand omics discovery. Teams are also continuing investigation of EEG biomarkers in the porcine model, astrocytic function in rodent experimental models, and blood‐based biomarkers in porcine and rodent experimental models and in humans.

#### 4.1.2 Future of the team science approach for PTE

The CURE Epilepsy PTE Initiative established a framework and networks to align preclinical and clinical investigators and develop tools and knowledge in animal and human PTE studies to enable the future identification of definitive biomarkers for PTE that may eventually aid in the development of therapeutic interventions. One advisor (Dr. Coulter) said “We are seeing the creation of models that can be mined for years to come”, whereas an investigator (Dr. Loeb) mentioned the value of “Developing data structures that can be used for this and other projects, specifically related to quantifying EEG recordings from animals and humans.”

The complex nature of PTE lent itself to a team science approach to advance the field. CURE Epilepsy's PTE Initiative was a catalyst in bringing together diverse teams of researchers providing cross‐collaboration and advancing the understanding of various facets of PTE and its pathophysiology.

In conclusion, a thorough understanding of the mechanisms leading to PTE and relevant biomarkers of epileptogenesis and PTE will require additional investment. While individual grants to explore this question are valuable, large collaborative efforts and consortia may have a bigger impact as they make use of collective wisdom and experience; such efforts would go a long way in uniting PTE investigators towards a common goal and in enabling collaboration.

## FUNDING INFORMATION

This work was supported by the Office of the Assistant Secretary of Defense for Health Affairs, through the Psychological Health and Traumatic Brain Injury Research Program under Award No. W81XWH‐15‐2‐0069.

## CONFLICT OF INTEREST STATEMENT

Neither of the authors has any conflict of interest to disclose. This work was supported by the Office of the Assistant Secretary of Defense for Health Affairs, through the Psychological Health and Traumatic Brain Injury Research Program under Award No. W81XWH‐15‐2‐0069. Opinions, interpretations, conclusions, and recommendations are those of the authors and are not necessarily endorsed by the Department of Defense. Drs. Churn, Umoh, and Whittemore were not involved in making funding decisions for the PTE Initiative.

## ETHICAL STATEMENT

We confirm that we have read the position of *Epilepsia Open* on issues involved in ethical publication and affirm that this report is consistent with those guidelines.

## Supporting information


Data S1.

## APPENDIX A

### A.1 CURE EPILEPSY POST‐TRAUMATIC EPILEPSY INITIATIVE ADVISORS (IN ALPHABETICAL ORDER)

Severn B. Churn^1^; Douglas Coulter^2^; Amanda Hunt^3^; Jaideep Kapur^4^; Daniel Lowenstein^5^; Renee Hebert Martin^6^; James McNamara^7^; Nsini Umoh^1^; H. Steve White^8^; Vicky Whittemore^1^; David W. Wright^9^

Advisor affiliations: ^1^National Institute of Neurological Disorders and Stroke; ^2^University of Pennsylvania; ^3^U.S. Department of Veterans Affairs; ^4^The University of Virginia Brain Institute; ^5^The University of California, San Francisco; ^6^Medical University of South Carolina; ^7^Duke University School of Medicine; ^8^University of Washington; ^9^Emory University School of Medicine.

### A.2 CURE EPILEPSY POST‐TRAUMATIC INITIATIVE INVESTIGATORS (IN ALPHABETICAL ORDER)

Brian Bacskai^1^; Michael Bambrick^1^; Jack L. Browning^2^; Mitchell Butler^3^; Rona Carroll^4^; Beth A. Costine‐Bartell^1^; Jared Davis^3^; Rossella Di Sapia^5^; Ann‐Christine Duhaime^1^; Joseph R. Geraghty^3^; Bryan Golemb^1^; Basso E. K. Gudenschwager^2^; James J. Gugger^6^; Mark Johnson^4^; Victoria Johnson^6^; Pavel Klein^7^; Firas Kobeissy^8^; Kyle Lillis^1^; Jeffrey A. Loeb^3^; Biswajit Maharathi^3^; Dzenis Mahmutovic^2^; Luis Martinez‐Ramirez^1^; Yara Mikhaeil‐Demo^9^; Federico Moro^5^; Kieran Normoyle^1^; Michelle L. Olsen^2^; Dipan C. Patel^2^; Rehan Raiyyani^1^; Teresa Ravizza^5^; Massimo Rizzi^5^; Stefanie Robel^10^; Anna Serafini^3^; Oleksii Shandra^2,11^; Harald Sontheimer^2^; Kevin Staley^1^; William Stewart^12^; Delia M. Talos^6^; Fernando Testai^3^; Michelle Theus^2^; Alexandra Ulyanova^6^; Pamela VandeVord^2^; Annamaria Vezzani^5^; Troy Volanth^2^; Amy K. Wagner^13^; Kevin K. W. Wang^8^; John Wolf^6^; Jensen Wong^3^; Elisa Zanier^5^

Investigator affiliations: ^1^Massachusetts General Hospital; ^2^Virginia Polytechnic Institute and State University; ^3^University of Illinois at Chicago; ^4^University of Massachusetts; ^5^Department of Acute Brain Injury, Mario Negri Institute for Pharmacological Research IRCCS; ^6^University of Pennsylvania; ^7^Mid‐Atlantic Epilepsy and Sleep Center; ^8^Morehouse School of Medicine; ^9^Northwestern Medicine; ^10^University of Alabama at Birmingham; ^11^Florida International University; ^12^University of Glasgow; ^13^University of Pittsburgh.
