# Supplementary material for: A team science approach for the preclinical and clinical characterization and biomarker development for post‐traumatic epilepsy
Source: Epilepsia Open. 2023 May 10;8(3):820–33. doi: 10.1002/epi4.12745 (PMC10472380; doi:10.1002/epi4.12745)
Supplement: Supplementary file 1 — Data S1. [file EPI4-8-820-s001.docx]

**Supporting Information 1: Logistics associated with the CURE Epilepsy Post-Traumatic Epilepsy (PTE) Initiative**

*Contracting and policies for the six selected teams*

The PTE Initiative was supported by the Office of the Assistant Secretary of Defense for Health Affairs, through the Psychological Health and Traumatic Brain Injury (TBI) Research Program and its associated grant, the Team Approach to the Prevention and Treatment of Post-Traumatic Epilepsy (TAPTE).^1^ Cooperative agreements were established between CURE Epilepsy and the Department of Defense (DoD), as well as subaward agreements between CURE Epilepsy and each of the six Principal Investigators (PIs) and institutions. All subawards were required to be approved by the DoD before execution.

*Personnel*

The structure of the PTE Initiative was built on CURE Epilepsy’s Infantile Spasms (IS) Initiative, taking into account successes such as the collaborative structure of the Initiative and the advisory structure.^2^ In addition, the PTE Initiative benefited from a set of key personnel with clearly defined roles. CURE Epilepsy’s PTE Initiative PI guided scientific direction and provided leadership, technical and financial oversight, and overall guidance to the internal CURE Epilepsy staff and the teams. One of the learnings from the IS Initiative was that enhanced management for a large-scale consortium is necessary,^2^ and thus a dedicated Project Manager and an Operations Assistant knowledgeable and experienced in implementing large research grants were added to the team. The role of the Project Manager (Author LHH) was to provide daily support and oversight for the Initiative. She served as the point person on review and submission of quarterly and annual reports and worked with teams and Advisors to address challenges as they were encountered. She was responsible for overseeing the planning of group meetings and communicated updates regarding the Initiative. She managed review of electronic case report forms for data standardization, managed the budget within CURE Epilepsy, and ensured release of payments to teams. As appropriate, she managed the creation and submission of no-cost extensions for the teams as well.

The strategic direction of the PTE Initiative was guided by the External Advisory Council (EAC) which evaluated the science, monitored progress by attending regular scientific progress meetings (which included an advisors-only discussion), provided advice between meetings, helped troubleshoot problems, offered solutions, suggested course changes, and provided input on sourcing supplies or recruiting clinical subjects. Members of the EAC also had input on when and how to terminate portions of projects. After the addition of the clinical projects to the Initiative, a Clinical Advisory Committee (CAC) was created along with the EAC. While the EAC primarily consisted of experts in preclinical epilepsy research, the CAC included experts on clinical TBI, statistics, and clinical epilepsy. Additionally, a DoD-appointed scientific officer liaised with CURE Epilepsy staff for all technical decisions related to DoD regulations and nuances and contributed to scientific oversight of the project by attending team meetings and providing feedback in the Advisors-only section of the meeting.

*Policies and agreements*

The IS Initiative revealed the need to develop policies in advance to adequately set expectations on confidentiality, publication, and data-sharing,^2^ and the PTE Initiative allowed teams to co-create standardized procedures and protocols with CURE Epilepsy. Policies created for the PTE Initiative included the Confidentiality Agreement, Publication Policy and Data Use and Sharing Agreement. Feedback from team members and advisors was sought over multiple rounds and was incorporated into policies before being finalized. An appeals process was instituted to enable formal decision-making regarding grievances and appeals of decisions made by the advisory councils and CURE Epilepsy. Policies and agreements developed as part of the PTE Initiative will be hosted on the CURE Epilepsy website ([www.cureepilepsy.org](http://www.cureepilepsy.org)).

*Reporting and auditing*

All teams were required to submit quarterly and annual reports to CURE Epilepsy and the DoD. The teams submitted reports to CURE Epilepsy, which were then reviewed and submitted to the DoD in combination with reporting of CURE Epilepsy’s activities. Financial and technical reporting were required and conformed to DoD templates.^3^

Each team had a Statement of Work (SOW) with clearly defined milestones, and CURE Epilepsy had an overall SOW that was approved by the DoD. The SOW included metrics of success that were discussed and agreed upon during the first meeting with advisors, grantees, and CURE Epilepsy; these were enforced and recalibrated if the need arose to “let the science lead the direction”. Adherence to the SOW for each team was discussed with CURE Epilepsy and the EAC. If a team did not meet its milestones within the stated amount of time, strategies were discussed to address any challenges. At times, there were deviations from milestones, and on these occasions, the rationale for deviation was discussed and guided by the EAC. For example, when an originally proposed animal model did not develop an expected rate of PTE, an alternative animal model was proposed and approved. Metrics of success were discussed

CURE Epilepsy also created an in-person auditing process to assess scientific and financial policies and procedures and the organization of each of the teams. In-person, day-long site visits to institutions where research was being performed were made by the PI of the Initiative and/or the Project Manager. Visits included a tour of facilities including laboratories, wet lab space, and animal facilities, and meetings with team members and institutional financial personnel. These visits were intended to ensure continued compliance and best practices. Areas of concern identified during these visits were noted on auditing checklists and then addressed to ensure a satisfactory resolution. Laboratory organization, experimental documentation, record security, experimental practices, and financial record-keeping were reviewed. To aid in these visits, auditing checklists were created that included details on organizational items (communication processes, managerial responsibilities), reporting and records management, and financial record-keeping.

*Data curation, storage, and sharing*

Following the identification by the EAC of a need for greater data standardization, curation, and storage, a partnership with the USC Laboratory of Neuroimaging (LONI) was instituted for the creation of online data standardization tools, e.g., electronic case report forms (eCRFs) containing common data elements (CDEs) and the creation of the data repository. Because a widely-used and vetted set of CDEs specific to PTE does not exist, CDEs contained in the eCRFs were sourced from epilepsy and TBI CDEs available on the NINDS CDE and Federal Interagency Traumatic Brain Injury Research Informatics System (FITBIR) websites.^4, 5^ Additionally, teams suggested several CDEs specific to their projects. eCRFs were made user-friendly (with drop-down tables, and auto-fillable fields) in collaboration with CURE Epilepsy CDE working groups and LONI. To accompany these data standardization tools, the CURE Epilepsy data repository was housed in The Image and Data Archive at the Laboratory of Neuro Imaging (LONI IDA). Although data sharing was not a prerequisite of each team’s grant, teams were encouraged to deposit data into the repository for cross-comparison and analysis and to preserve the longevity of the data.

*Progress meetings*

While CURE Epilepsy’s earlier IS Initiative led to many successes, one lesson learned was the need for proactive planning to ensure that PIs and early career team members could effectively share data and ideas.^2^ Hence, meetings for the PTE Initiative were planned for an entire year in advance to manage scheduling challenges. Two annual face-to-face meetings featured updates from all six teams as well as an advisors-only session to discuss progress and challenges. At times, these in-person meetings featured workshops and special guest speakers from outside the Initiative who spoke on a variety of topics such as the creation of tools for the standardization of preclinical epilepsy research or applying artificial intelligence and machine learning to PTE research. Points of collaboration were often discovered during these meetings which led to separate research projects. A few examples of tangible collaborations as a result of the progress meetings were sharing of electroencephalogram (EEG) algorithms between The University of Illinois at Chicago, Mario Negri Institute for Pharmacological Research, and Virginia Polytechnic Institute and State University teams, and those where omics protocols were shared between the University of Florida and the Mid-Atlantic Epilepsy and Sleep Center (MAESC) teams. Before the COVID-19 pandemic, there were quarterly virtual meetings, which enabled a seamless transition of meeting operations during the pandemic. Additionally, during shorter bi-monthly meetings, early-career investigators were encouraged to present short updates on their findings, to promote engagement and recognition of the entire team. These meetings were vital to ensure collaboration between teams.

**Supporting Information 2: Focus groups formed as part of the CURE Epilepsy PTE Initiative**

The EEG Focus Group: Meetings in this focus group came from the recognition of the lack of standards for visualizing and analyzing EEG, both within the Initiative and in the field of PTE research. Despite the availability of standardized EEG terminology and definitions from fields outside of PTE and the utility of EEG in preclinical and clinical epilepsy research, there is a lack of high-quality, evidence-based guidelines and definitions for the clinical use of EEG specifically in TBI and PTE patients. During EEG focus group meetings, team members presented their EEG findings, and methods of visualization and analysis, discussed terminology, and compared guidelines in preclinical models to clinical criteria. The goals were to develop a standardized definition of epileptiform activity and identify EEG signatures indicative of the development of PTE (EEG biomarker) and seizures before PTE has developed. The role of the EEG as a translational linkage between animal and human studies was discussed as well. Differences and similarities in EEG patterns and their meanings, the importance of videos, montage, time of recordings (acute vs. chronic), and comparisons to human EEGs were also part of this focus group. The value of EEG algorithms in areas such as spectral analysis along with ictal and inter-ictal discharge detection were also discussed. Common EEG artifacts from all models were presented and discussed, to define not only what is important for PTE, but also what is not PTE, as oftentimes, some EEG artifacts may appear as seizures or epileptiform abnormalities. These meetings served as a source of knowledge and training for early career investigators and was a key part of their training in epilepsy research. These investigators took on the responsibility of developing, distributing, and reviewing questionnaires to all teams to identify commonalities and differences in their TBI and EEG acquisition systems and parameters.

The Data Sharing Focus Group: This group arose from the need to better define the data to be put into the University of Southern California Laboratory of Neuro Imaging Image and Data (LONI) repository and focused on the sharing of both clinical and preclinical data. The group appraised the clinical data available and defined the most clinically-relevant preclinical variables to be shared and entered into the LONI Image and Data Archive (IDA) repository. The group also discussed what comparisons might be made given the variety of animal injury models, human populations, and time points.

Due to institutional delays in data use agreement negotiations, and the fact that data use agreements were not built into clinical study consent forms from the start, the aim of having clinical data shared and stored through the repository was not feasible, representing a learning opportunity. Instead, efforts were focused on the sharing of preclinical data and utilization of shared algorithms to compare the development of epileptiform activity and changes in omic signatures post-TBI. This goal, too, was hampered by issues such as difficulties converting files into a required EEG file format for EEG data upload, representing a technical hurdle to be overcome.

Clinical and preclinical CDE Focus Group: This group was created to discuss CDEs and develop CRFs for preclinical and clinical TBI/epilepsy research. Information from publicly-available NINDS CDE and FITBIR websites,^4, 5^ which contain CDEs on information such as species and basic animal information, genetic background, variables specific to different injury types (i.e., CCI, weight drop, etc.), and clinical information were leveraged. Several CDEs were also defined by the focus group to fill gaps about variables relevant to the porcine CCI model. Once variables were identified and created, CDEs were converted into an electronically fillable form by LONI. The goals were 1) to create a set of user-friendly forms for data capture and reporting to improve the rigor and transparency of PTE Initiative studies, and 2) to allow investigators to provide a record of the data to be deposited into the LONI repository.

**References:**

1. Epilepsy ICtARi. Team approach to the prevention and treatment of post-traumatic epilepsy (TAPTE). Available at: <https://icarerp.nih.gov/project/team-approach-prevention-and-treatment-post-traumatic-epilepsy-tapte-0>. Accessed November 30, 2021.

2. Lubbers L, Iyengar SS. A team science approach to discover novel targets for infantile spasms (IS). Epilepsia Open. 2021;6:49-61.

3. Traumatic Brain Injury and Psychological Health Research Program (TBIPH): Electronic Biomedical Research Application Portal (eBRAP). Available at: <https://ebrap.org/eBRAP/public/ProgramFY.htm?programFYId=374901>. Accessed August 8, 2021.

4. Preclinical traumatic brain injury common data elements. Available at: <https://fitbir.nih.gov/content/preclinical-common-data-elements>. Accessed August 13, 2022.

5. (NINDS) NIoNDaS. NINDS Common Data Elements: Harmonizing information. Streamlining research. Available at: <https://www.commondataelements.ninds.nih.gov/>. Accessed November 30, 2021
